# Dysbiosis of the oral-gut microbiome axis in a mouse model of depression

**DOI:** 10.1371/journal.pone.0355302

**Published:** 2026-08-11

**Authors:** Pu Lei, Shaonan Tong, Wenyu Xi, Yixin Liu, Jiayue Yu, Lu Yu, Binbin Zhao, Min Jia, Ye Li, Xiancang Ma, Yunpeng Wang, Yijie Guo

**Affiliations:** 1 Department of Psychiatry, The First Affiliated Hospital of Xi’an Jiaotong University, Xi’an, China; 2 Med-X Institute, Center for Immunological and Metabolic Diseases, The First Affiliated Hospital of Xi’an Jiaotong University, Xi’an, China; 3 Center for Translational Medicine, The First Affiliated Hospital of Xi’an Jiaotong University, Xi’an, China; 4 Clinical Research Center of Shaanxi Province for Dental and Maxillofacial Diseases, College of Stomatology, Xi’an Jiaotong University, Xi’an, China; 5 Center for Brain Science, The First Affiliated Hospital of Xi’an Jiaotong University, Xi’an, China; University of Minnesota, UNITED STATES OF AMERICA

## Abstract

This study aimed to characterize the alterations in both oral and gut microbiota in a mouse model of depression and to explore their potential role in the pathogenesis of major depressive disorder (MDD) through the oral-gut-brain axis. A depression model was established in male C57BL/6J mice using chronic social defeat stress (CSDS) paradigm. Depressive phenotypes were confirmed through social interaction, sucrose preference, open field, tail suspension, and forced swim tests. The microbial composition of oral and gut samples was analyzed using 16S rRNA sequencing, with Linear Discriminant Analysis Effect Size (LEfSe) employed to identify differentially abundant taxa and Spearman correlation analysis to examine microbiota-behavior relationships. CSDS successfully induced robust depression-like behaviors, including social avoidance, anhedonia, and behavioral despair. Beta-diversity analysis revealed significant separation in oral microbiota between CSDS and control groups. LEfSe analysis identified distinct microbial signatures: control mice were enriched in oral *Streptococcus* and gut commensals including *Lachnospiraceae*, *Bacteroides* and *Oscillospiraceae*, whereas CSDS mice showed expansion of oral *Muribacter* and *Rodentibacter* and gut *Alloprevotella*, *Helicobacter* and *Colidextribacter*. Correlation analyses demonstrated significant associations between specific microbial patterns and depression-like behaviors, with control-enriched taxa negatively correlating with behavioral deficits. Furthermore, significant cross-habitat microbial correlations were observed between oral and gut differential taxa. Our findings demonstrate that CSDS induces divergent microbial alterations in both oral and gut ecosystems, which are systematically associated with depression-like behaviors. These results provide compelling evidence for the involvement of the oral-gut-brain axis in depression pathophysiology and suggest that modulating these microbial ecosystems may represent a potential therapeutic strategy for MDD.

## Introduction

Major depressive disorder (MDD) is a prevalent and severe neuropsychiatric condition, affecting millions of individuals worldwide and imposing a substantial global health burden [1]. The clinical presentation of MDD is characterized by persistent low mood, anhedonia, feelings of hopelessness, and sleep disturbances, which profoundly diminish the quality of life of affected individuals [2–5]. Despite its significant prevalence and impact, the precise pathogenesis of MDD remains elusive. Prevailing theories have implicated a range of factors, including hippocampal atrophy, dysregulation of the hypothalamic-pituitary-adrenal (HPA) axis immune functions [6–8]. However, anti-depressant therapies grounded in these established hypotheses alleviate symptoms in only approximately half of all patients [9], this underscores the urgent need to explore novel mechanistic perspectives.

In recent years, the gastrointestinal tract, housing millions of microorganisms collectively termed the gut microbiome, has emerged as a critical regulator of brain function and behavior via the microbiota-gut-brain (MGB) axis [10]. Compelling evidence from both human and animal studies has demonstrated significant shifts in the gut microbial composition of patients with MDD, characterized by alterations in key bacterial taxa such as enriched *Alistipes* and depleted *Faecalibacterium* [11,12]. Fecal microbiota transplantation from patients with MDD to germ-free mice can induce depressive-like behaviors [13]. Similarly, transplantation of microbiota from mice subjected to chronic social defeat stress is sufficient to transfer depressive phenotypes to recipient animals [14]. Collectively, these findings support a pivotal role for gut dysbiosis in depression.

Parallel to the gut, the oral cavity, harboring the second most diverse microbial community in the human body, is increasingly recognized for its systemic implications. The “oral-gut axis” illustrates how oral microbial homeostasis profoundly influences distal sites, with oral bacteria serving as a reservoir for potential gut pathobionts [15]. Emerging clinical studies have begun to link the composition of the oral microbiome to the occurrence of depression [16,17]. While these findings highlight the potential role of the oral microbiome, our understanding of its specific mechanisms in depression remains limited.

Chronic social defeat stress (CSDS) is a well-established rodent model for studying depression-like behaviors, effectively recapitulating core symptoms such as social avoidance, anhedonia, and behavioral despair [18]. In this study, we established a CSDS-induced depression model in mice and performed 16S rRNA sequencing to systematically compare the structural differences in oral and gut microbiota between stressed and control groups. Furthermore, differential bacterial genera between the CSDS and control groups in both the oral and gut environments were identified using linear discriminant analysis. By analyzing both oral and gut microbiota and connecting them to depression-like behaviors, we provide new evidence for the oral-gut-brain axis in MDD. Our work lays a foundation for future studies on this systemic connection.

## Materials and Methods

### Mice

Male C57BL/6 mice (aged 8−10 weeks) and male CD-1 ICR mice (aged 12−16 weeks) were obtained from the Experimental Animal Center of Xi’an Jiaotong University Medical College and maintained in a temperature-controlled (20–22°C; 45–65% humidity; 12:12 h light:dark cycle) specific-pathogen-free (SPF) level environment. The mice were given water and commercial standard feed *ad libitum*. All experimental procedures were performed in accordance with the guidelines of the Institutional Animal Care and Use Committee of Xi’an Jiaotong University. The protocol was approved by the Committee on the Ethics of Animal Experiments of the Xi’an Jiaotong University (Protocol Number: XJTUAE2024−493). Animals were anesthetized using isoflurane (induction 4%, maintenance 2%) in oxygen, with loss of toe pinch reflex confirming surgical anesthesia. To minimize suffering, all procedures were performed under deep anesthesia, and animals did not recover from anesthesia. Animals were euthanized by an overdose of isoflurane (5% for 5 minutes) followed by cervical dislocation, and death was confirmed by cessation of heartbeat and breathing.

### Chronic social defeat stress (CSDS)

CD-1 mice were used to screen for aggressors. C57BL/6 mice were randomly divided into a control group (n = 10) and a CSDS group (n = 10) and underwent the CSDS paradigm according to the standard protocol [19,20]. Each mouse in the CSDS group was defeated by an aggressive CD-1 mouse for 5 min in one side of the cage, and then transferred to the other side to experience sensory stress for 24 h. The CSDS paradigms lasted for 14 consecutive days. Control mice were housed in identical two-compartment cages without an aggressor CD-1 mouse, and were handled similarly but not subjected to any defeat or sensory stress. Body weight was measured before and after the CSDS paradigm.

### Social interaction (SI) test

The SI test was conducted on day 10. Briefly, each mouse was placed in an open arena (40 × 40 × 50 cm) with an interaction box on one side. The test consists of two 150 second trials: first, without (no-target) and then with (target) an unfamiliar male CD-1 mouse inside the box. The time spent in a 7 cm wide interaction zone surrounding the box was measured for each trial. The SI ratio was calculated as follows: (time in zone with target – time in zone without target)/ (time in zone with target + time in zone without target). Mice with an SI ratio below 1 were defined as susceptible.

### Behavioral tests

After the last CSDS session, all control and CSDS mice were singly housed with an interval of 24 h and then subjected to a behavioral testing battery to evaluate depressive-like behaviors. Analyses were performed in a manner blinded to treatment assignments in all behavioral experiments. The duration and entries in the open field test (OFT) to the designated area, as well as the immobile time in the tail suspension test (TST) and the forced swim test (FST) of all mice, were quantified using the automated video-tracking software SMART 3.0 (Panlab SL, Spain).

### OFT

The OFT assessed locomotion in a novel environment. Mice were placed individually in an open-field box (45 × 45 × 45 cm) and allowed to explore freely for 30 min. The distance traveled, the time spent, and the number of entries into the central zone (15 × 15 cm) were measured [21].

### TST

The mice were suspended by their tails 60 cm above the floor, in a white plastic chamber. Adhesive tape was placed less than 1 cm from the tip of the tail. Their behavior was recorded for 10 min using a video camera, and the immobility time was measured. In this test, the immobility was defined as the period when the animals stopped struggling for more than 1 second [22].

### FST

The mice were placed individually in Plexiglass cylinders (30 cm in height and 15 cm in diameter) filled with 15 cm of water (25 ± 1°C). Test sessions lasted for 6 min, with the last 5 min scored for immobility. A mouse was judged to be immobile when it remained floating in an upright position, making only the movements necessary to keep its head above the water [23].

### SPT

Decrease in sucrose preference is generally considered an indicator of anhedonia, which is a core symptom of depression [24]. Prior to the test, all the mice were trained to consume 1% sucrose solution for two days. On the third day, an SPT was conducted. The mice were given free access to one bottle containing 100 mL of water and another bottle containing 100 mL of 1% sucrose solution. The positions of the two bottles were alternated every 12 h to avoid position preference. At the beginning and end of the 24 h test, the solution weights were measured, and the sucrose preference was calculated as: sucrose consumption/ (water consumption + solution consumption) × 100%.

### Sample collection

Mice were sacrificed after the last behavioral test and oral samples were collected using cotton swabs from the back of the tongue to the palate, buccal mucosa, upper and lower vestibules, and floor of the mouth for 30 s. Fresh fecal samples from the colon of each mouse were collected using sterile cotton swabs, placed in a sterile sampling tube, and quickly transferred to a −80°C freezer.

### High-throughput 16S rRNA gene amplicon sequencing

Total genomic DNA was extracted from saliva and stool samples using the TGuide S96 Magnetic Stool DNA Kit (Tiangen Biotech, Beijing, China) according to manufacturer’s instructions. DNA quality and quantity were assessed via electrophoresis on a 1.8% agarose gel and measured using a NanoDrop 2000 UV-Vis spectrophotometer (Thermo Scientific, Wilmington, USA). The V3–V4 hypervariable region of the bacterial 16S rRNA gene was amplified using primers 338F (5‘-ACTCCTACGGGAGGCAGCA-3’) and 806R (5‘-GGACTACHVGGGTWTCTAAT-3’). Both forward and reverse primers were tailed with sample-specific Illumina index sequences. PCR was performed in a 20 μL reaction volume containing 5–50 ng DNA template, 0.3 μL of each primer (10 μM), 5 μL KOD FX Neo Buffer, 2 μL dNTP (2 mM each), 0.2 μL KOD FX Neo, and ddH_2_O to final volume. The thermal cycling protocol consisted of initial denaturation at 95 °C for 5 min; 20 cycles of denaturation at 95 °C for 30 s, annealing at 50 °C for 30 s, and extension at 72 °C for 40 s; and a final extension at 72 °C for 7 min. Amplified products were purified using an Omega DNA Purification Kit (Omega Inc., USA) and quantified using Qsep-400 (BiOptic, Inc., Taiwan). Paired-end sequencing (2 × 250 bp) was performed on an Illumina NovaSeq 6000 platform (Beijing Biomarker Technologies Co., Ltd., China).

### Bioinformatics analysis

Raw sequencing reads were demultiplexed and quality-filtered using the DADA2 pipeline within QIIME 2 (2020.11). Specifically, reads were trimmed, denoised, merged, and chimeras were removed to generate amplicon sequence variants (ASVs). ASVs with a total count of less than 2 across all samples were filtered out. Taxonomy assignment was performed using the Naive Bayes classifier in QIIME 2 against the SILVA database (release 138.1) with a confidence threshold of 70%. Alpha diversity (Shannon and Simpson indices) was calculated using QIIME 2 and visualized with R software. Beta diversity was assessed using two complementary distance metrics: Jaccard distance (presence/absence-based) and Bray-Curtis distance (abundance-weighted). Dissimilarity matrices were visualized via non-metric multidimensional scaling (NMDS). Permutational multivariate analysis of variance (PERMANOVA) was used to test for significant differences between groups. Linear discriminant analysis Effect Size (LEfSe) was performed to identify differentially abundant genera among the four groups (Con-O, CSDS-O, Con-G, CSDS-G). A logarithmic LDA score of 4.0 was set as the threshold for discriminative features. For correlation analyses (behavior-microbiota and oral-gut microbiota), Spearman’s rank correlation coefficient was calculated using R software (psych package), and heatmaps were generated using the pheatmap package.

### Statistical analysis

Data analysis and graphing of behavioral data were performed using GraphPad Prism 8.0. All continuous variables, including behavioral data and bacterial alpha diversity indices, were presented as means ± SEM, unless otherwise indicated, and compared between groups using Student’s t test or one-way ANOVA as appropriate. Statistical significance was set at *P* < 0.05.

### Ethics declarations

All procedures involving mice experiment in this study comply with the ethical standards of the institution research ethics committee and the Helsinki Declaration. The studies involving mice experiment were reviewed and approved by The Ethics Committee of the First Affiliated Hospital of Xi’an Jiaotong University. Clinical trial number: not applicable.

## Results

### CSDS induces robust depression-like behaviors in mice

**Fig 1:**
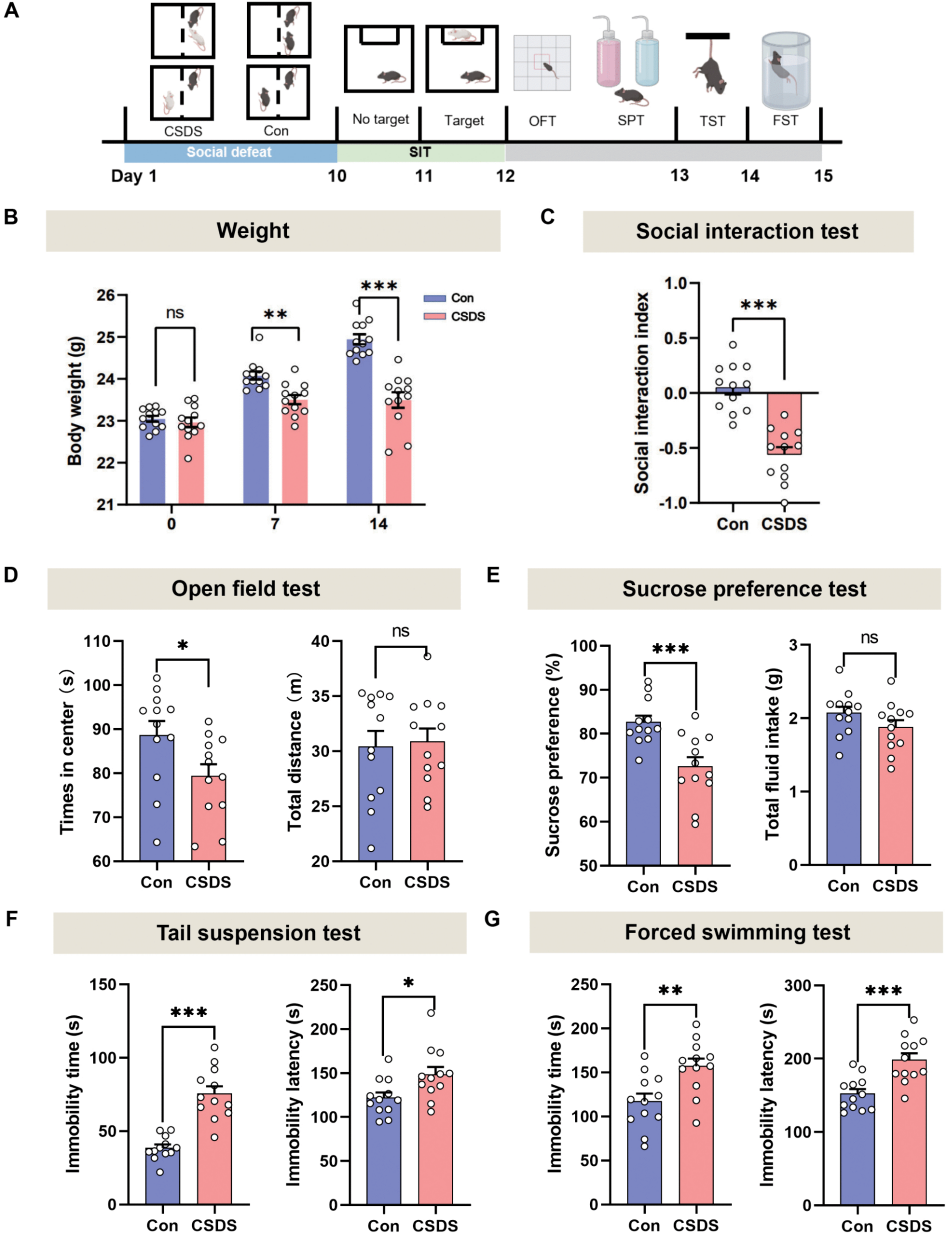
CSDS induces depression-like phenotypes in mice. (A) Experimental timeline of the chronic social defeat stress (CSDS) paradigm and subsequent behavioral tests. (B) Body weight of mice measured on day 0 and 10. (C) Summary plots of the social interaction (SI) ratio of control and CSDS mice. (D-G) Behavioral tests of CSDS-susceptible mice compared to controls, including: total distance and time spend in the center of OFT (D); the percentage of sucrose intake and total fluid intake in SPT (E); the immobility time and latency in TST (F) and in FST (G). (n = 10, * *P* < 0.05, ** *P* < 0.01, *** *P* < 0.001).

The experimental timeline is shown in Fig 1A. Prior to the initiation of CSDS paradigm (day 0), there was no statistically significant difference in body weight between the two groups of mice. Following 10 days of CSDS exposure, however, the body weight of mice in the CSDS group was significantly reduced compared to that in the control group (Fig 1B). In the SI test, the mean social interaction ratio of CSDS-exposed mice was significantly lower than that of control mice (−0.561 ± 0.07 vs. 0.048 ± 0.06), indicating robust social avoidance behavior in the stress-exposed cohort (Fig 1C). In the OFT, the time spent in the central zone in CSDS-exposed mice was significantly shorter than that of control mice. In contrast, no significant difference in total distance traveled was observed between the two groups (Fig 1D). These findings confirmed that CSDS selectively induced anxiety-like behavior in mice without compromising general motor function. In the SPT, CSDS-exposed mice showed a significant decrease in sucrose preference, while total fluid intake remained comparable between the two groups (Fig 1E). This result suggested that CSDS induced anhedonia in mice without altering physiological thirst drive or basic drinking capacity. Notably, in both the TST and FST, CSDS-exposed mice exhibited significantly prolong immobility time and shortened latency to immobility relative to control mice (Fig 1F and 1G), suggesting a prominent state of behavioral despair. Collectively, these behavioral findings demonstrated that our CSDS paradigm induced a robust depressive-like phenotype in mice, characterized by social avoidance, anhedonia, anxiety-like behavior, and behavioral despair. These convergent results validated the successful establishment of the mouse model of depression.

### Analysis of alpha and beta diversity in oral and gut microbiota of CSDS mice

**Fig 2:**
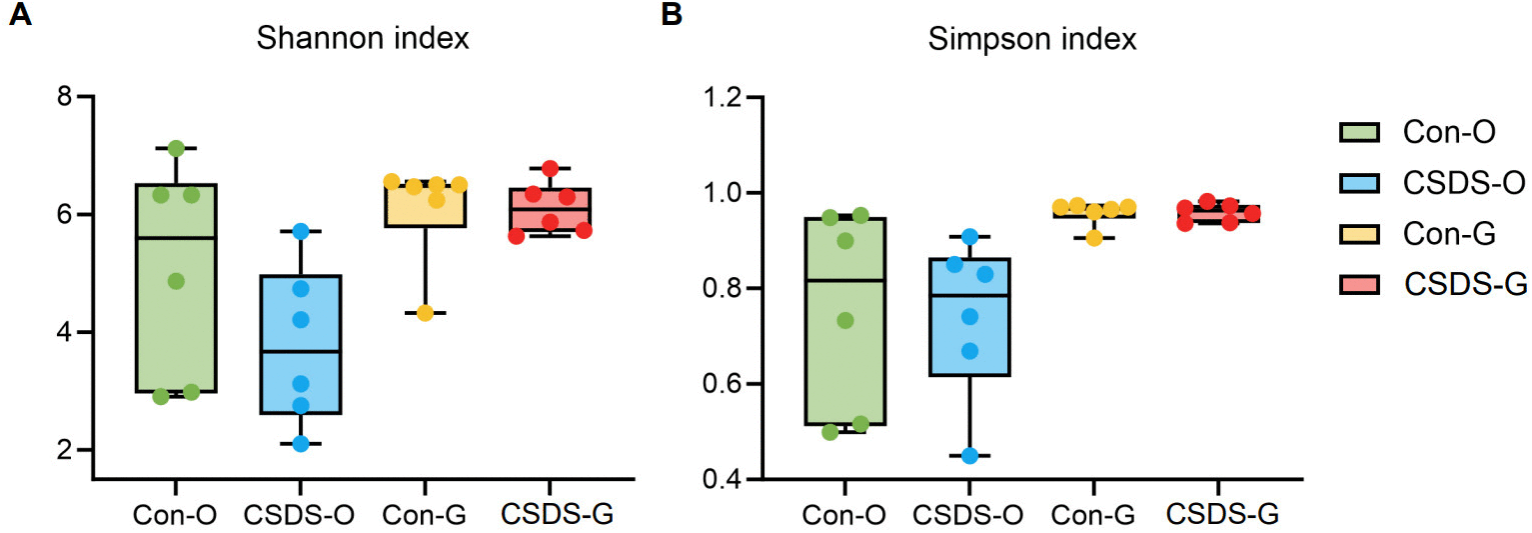
Difference of alpha diversity in healthy and CSDS-induce depression mice. (A) Differences in alpha diversity of oral-gut flora in the two groups using Shannon index. (B) Differences in alpha diversity of oral-gut flora in the two groups using Simpson index. O stands for oral flora, G stands for gut flora; Con stands for Healthy mice, CSDS stands depression mice (n = 6). For 16S rRNA sequencing, DNA extraction from oral samples was challenging due to the relatively low microbial biomass in the mouse oral cavity. After stringent quality control (DNA concentration and purity), 6 samples per group that met the criteria were included in the final microbiome analysis.

To investigate the potential impact of depression on oral and gut microbiota, we collected saliva and fecal samples from CSDS mice and performed 16S rRNA sequencing. Alpha diversity was assessed using the Shannon index (Fig 2A) and Simpson index (Fig 2B). No significant differences in either index were observed between the CSDS and control groups within the oral microbiota (CSDS-O vs. Con-O) or within and gut microbiota (CSDS-G vs. Con-G). However, regardless of group, the gut microbiota exhibited higher alpha diversity compared to the oral microbiota. These findings suggest that the oral cavity harbors a less diverse community than the gut in this mouse model，while chronic stress did not significantly alter alpha diversity in either habitat.

**Fig 3:**
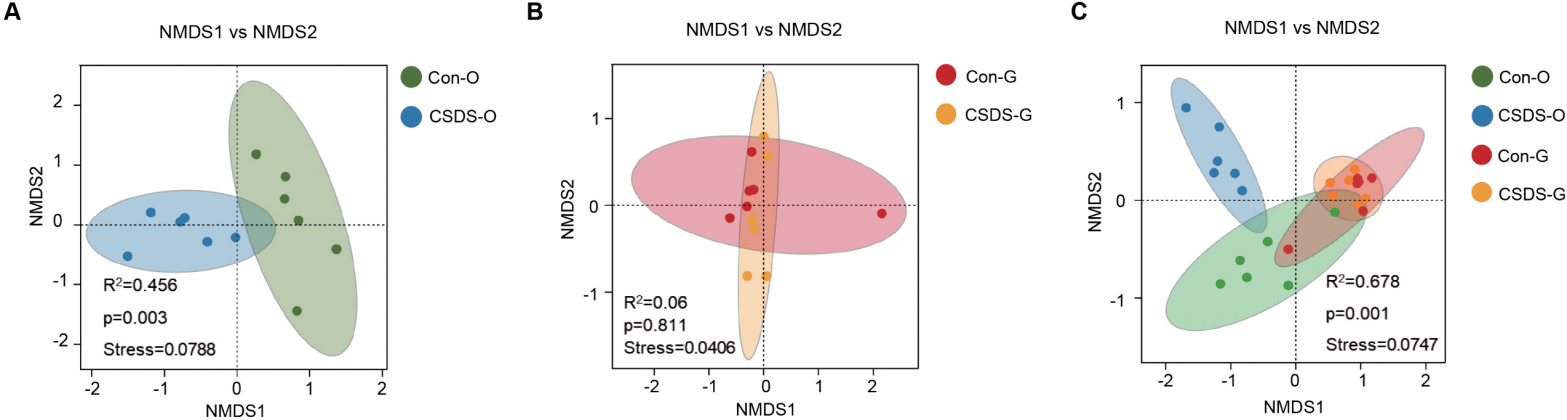
Difference of beta diversity in healthy and CSDS-induce depression mice. (A) Difference of beta diversity in oral flora, (B) Difference of beta diversity in gut flora, (C) NMDS plot showing the overall separation between oral and gut microbial communities (Con-O, CSDS-O, Con-G, CSDS-G) based on Jaccard distance. This panel is presented for qualitative visualization only; no direct statistical comparison was performed between oral and gut groups. O stands for oral flora, G stands for gut flora; Con stands for Healthy mice, CSDS stands depression mice (n = 6).

We assessed beta diversity of the oral and gut microbial communities using NMDS based on Jaccard distance at the ASV level. The analysis revealed a significant separation in the oral microbiota between the CSDS and control groups (R² = 0.456, *p* = 0.003; Fig 3A), indicating a robust stress-induced alteration in oral microbial composition. In contrast, the gut microbiota did not exhibit significant separation between the two groups (Fig 3B). Moreover, the NMDS plot showed a clear distinction in overall microbial community structure between the oral and gut environments (Fig 3C). Consistent results were obtained using abundance-weighted Bray-Curtis distance (S1 Fig), confirming that the CSDS-induced oral microbial shift is robust to both presence/absence and abundance-based metrics.

### Analysis of Oral and Gut Microbiota Composition in the CSDS mice

**Fig 4:**
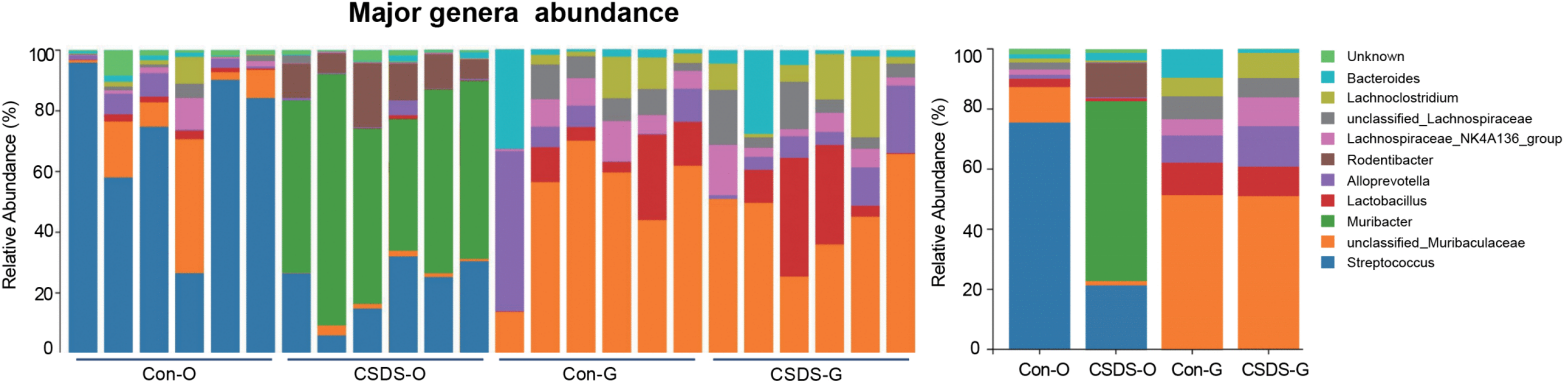
The composition and relative abundance of oral and gut flora at the genus level. Bar plots displaying taxonomic composition at the major genera of each sample (left) and average abundance in the Con-O, CSDS-O, Con-G, CSDS-G groups (right). No direct statistical comparisons were made between oral and gut habitats; the purpose of this side-by-side display is to identify genera that may be involved in oral-gut crosstalk under stress. O stands for oral flora, G stands for gut flora; Con stands for Healthy mice, CSDS stands for depression mice.

To delineate the alterations in microbial composition at the genus level among the four groups, we profiled and compared their microbial structures. As shown in Fig 4, the oral microbiota underwent substantial changes following CSDS intervention. The three most abundant genera in the oral cavity were *Streptococcus* (47.0% in Con-O vs. 17.3% in CSDS-O), *unclassified Muribaculaceae* (7.4% vs. 1.2%), and *Muribacter* (0.003% vs. 48.4%). Notably, the CSDS-O group exhibited a marked reduction in the relative abundances of *Streptococcus* and *unclassified Muribaculaceae*, whereas genera such as *Muribacter* and *Rodentibacter* were significantly enriched. These collective shifts suggest a substantial disruption of the oral microbial ecosystem induced by CSDS.

In contrast, the gut microbiota exhibited more subtle compositional changes. The three most abundant genera were *unclassified Muribaculaceae* (24.5% in Con-G vs. 25.2% in CSDS-G), *Lactobacillus* (5.2% vs. 4.8%), and *Alloprevotella* (4.3% vs. 6.6%). Compared to the Con-G group, the CSDS-G group showed a marked depletion of *Lactobacillus*, alongside a significant enrichment of *Alloprevotella*, indicating a state of microbial dysbiosis. In summary, our genus-level analysis reveals that CSDS intervention induced distinct microbial dysbiosis in both oral and gut ecosystems, with the alterations being substantially more pronounced in the oral cavity.

### LEfSe Analysis of Oral and Gut Microbiota in the CSDS mice

**Fig 5:**
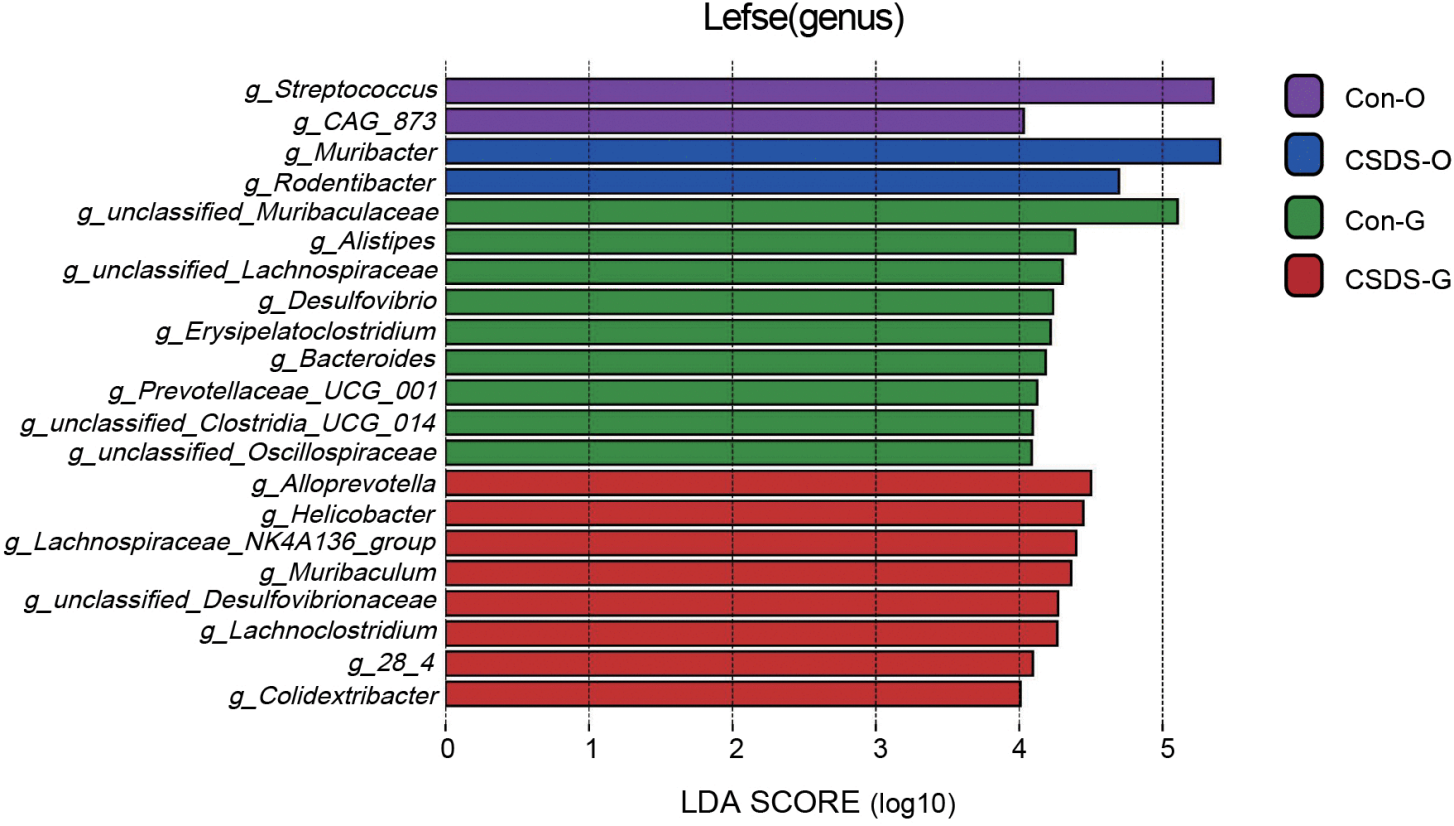
Taxonomic biomarkers found by LEfSe in each of the four groups. While the figure presents all four groups simultaneously, the statistical comparisons were performed separately within each habitat (oral CSDS vs. oral control; gut CSDS vs. gut control). No cross-habitat comparisons (e.g., Con-O vs. Con-G) were tested. O stands for oral flora, G stands for gut flora; Con stands for Healthy mice, CSDS stands for depression mice.

Linear discriminant analysis Effect Size (LEfSe) was employed to identify statistically significant biomarkers discriminate the microbial communities among the four groups (Con-O, CSDS-O, Con-G, CSDS-G). The analysis revealed distinct, group-specific microbial signatures, as illustrated in the LDA bar plot (Fig 5).

In the oral microbiota, the Con-O group was characterized by a higher abundance of genera such as *Streptococcus* and *CAG_873*. In contrast, the CSDS-O group exhibited significant enrichment of taxa including *Muribacter* and *Rodentibacter*, which have been previously linked to an inflammatory oral environment [16,25]. This marked shift indicates a stress-induced dysbiosis in the oral cavity.

Within the gut microbiota, the Con-G group showed a predominance of commensal genera, including *unclassified Muribaculaceae*, *Alistipes*, *unclassified Lachnospiraceae*, *Desulfovibrio*, *Erysipelatoclostridium*, *Bacteroides*, *Prevotellaceae_UCG_001*, *unclassified Clostridia_UCG_014*, and unclassified *Oscillospiraceae*. Many of these are recognized for their roles in maintaining gut homeostasis and producing short-chain fatty acids (SCFAs) [26–32]. Conversely, the CSDS-G group demonstrated robust enrichment of genera such as *Alloprevotella*, *Helicobacter*, *Lachnospiraceae_NK4A136_group*, *Muribaculum*, *unclassified Desulfovibrionaceae*, *Lachnoclostridium*, *28_4*, and *Colidextribacter*. Collectively, these LEfSe results demonstrate that CSDS intervention induces distinct, ecosystem-specific dysbiosis patterns in both the oral and gut microbiota.

### Correlation Analysis Between Oral and Gut Differential Microbiota and Depressive-Like Behavior

**Fig 6:**
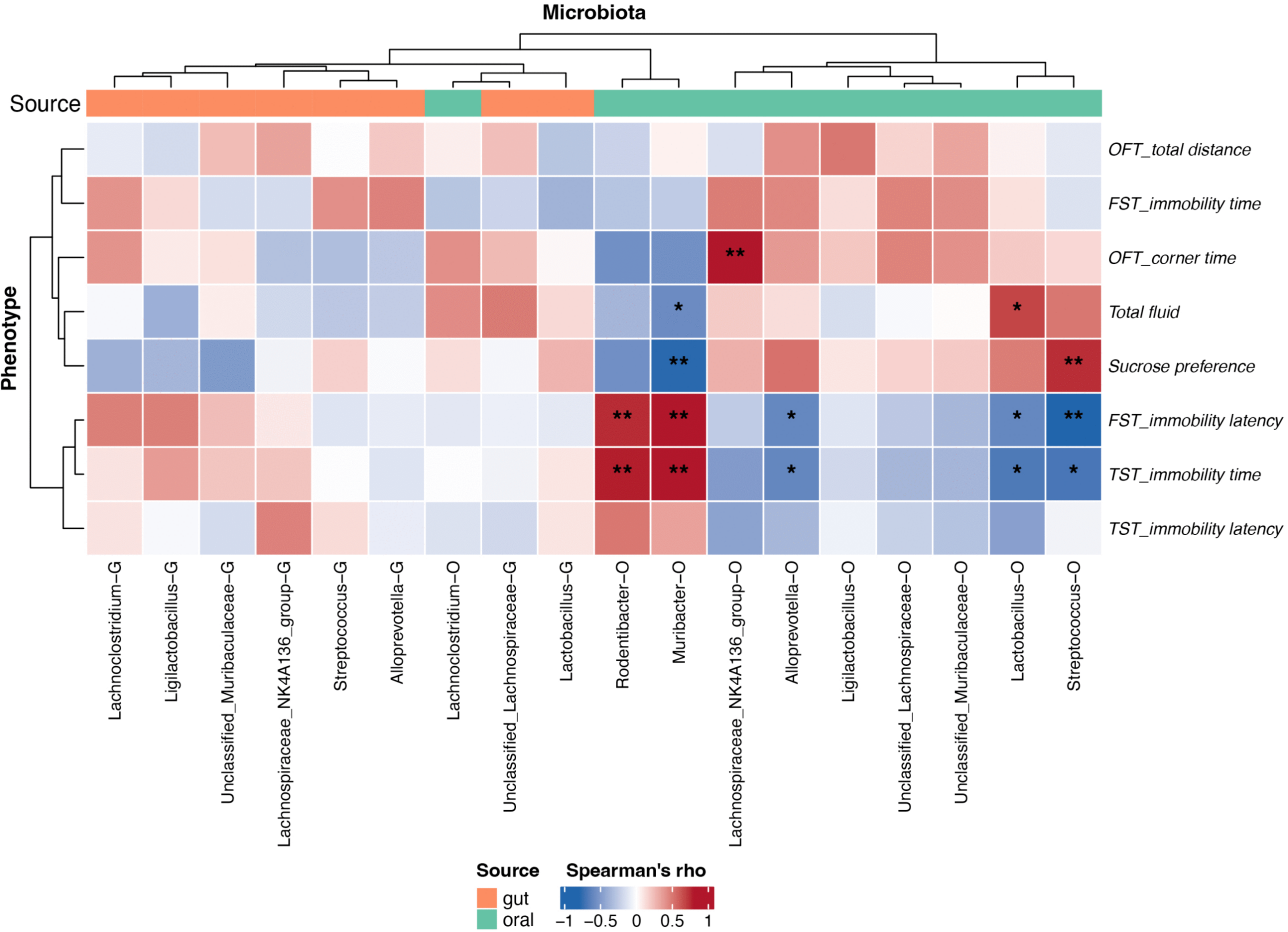
Heatmap of correlation between oral-gut differential flora and depressive-like behavior. Red indicates positive correlation and blue indicates negative correlation (*P < 0.05, **P < 0.01, ***P < 0.001).

To evaluate the relationship between differential microbiota and depressive-like behavior, spearman correlation analysis was performed. The results revealed the genera enriched in control mice, including *Streptococcus-O* and *Lactobacillus-O*, were inversely associated with behavioral despair measures, showing significant negative correlations with both TST immobility and FST latency. Notably, *Streptococcus-O* abundance was also positively correlated with sucrose preference. A parallel but non-significant trend was also observed for *unclassified Muribaculaceae-O* genus. In contrast, taxa expanded in CSDS mice, including *Muribacter-O* and *Rodentibacter-O*, displayed significant positive correlations with TST immobility and FST latency, while *Muribacter-O* also showing a negatively correlation with sucrose preference (Fig 6). These results collectively suggest that specific alternations in the microbiota are closely associated with the expression of depressive-like behaviors.

### Correlation Analysis Between Oral and Gut Differential Microbiota

**Fig 7:**
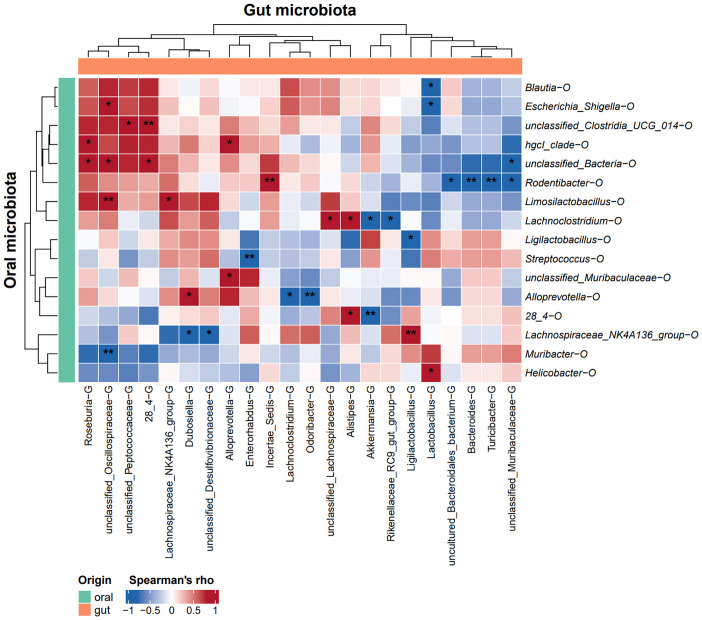
Heatmap of correlation between oral and gut microbiota. Red indicates positive correlation and blue indicates negative correlation (*P < 0.05, **P < 0.01, ***P < 0.001).

Spearman correlation analysis of oral and gut differential microbiota revealed a significant inverse association between taxa enriched in control group and those expanded in the CSDS group. For instance, *unclassified Muribaculaceae-G* exhibited negative correlations with *Rodentibacter-O* and *Alloprevotella-O*. Similarly, *Bacteroides-G* was negative correlated with *Rodentibacter-O*, and *unclassified Oscillospiraceae-G* showed a negative correlation with *Muribacter-O*. Furthermore, *Rodentibacter-O* was inversely associated with *Turicibacter-G*. In contrast, positive correlations were observed between *unclassified Lachnospiraceae-G* and *Lachnoclostridium-O*, as well as between *Alistipes-G* and *Lachnoclostridium-O* (Fig 7). These coordinated correlation patterns suggest potential ecological interactions between microbial taxa across different habitats in the context of depression.

## Discussion

Collective evidence indicates that the oral microbiota undergoes specific, albeit model-dependent, dysbiosis in response to psychological stress. In the present study, CSDS induced distinct alterations in the oral microbial community. A key finding was the marked reduction of *Streptococcus* and *unclassified Muribaculaceae* in the oral cavity of CSDS mice. These taxa are recognized as core commensals in the oral ecosystem, with *Streptococcus* species playing crucial roles in maintaining microbial homeostasis and modulating oral immune balance [33]. The depletion of oral *Streptococcus* in our CSDS model aligns with its identification as a dominant genus in healthy control groups in previous tooth loss study, reinforcing its role as a key commensal microbiota in oral homeostasis [34].

Conversely, the CSDS group exhibited a pronounced expansion of genera such as *Muribacter* and *Rodentibacter*. This observation is consistent with reports from chronic restraint stress (CRS) models, which similarly documented the enrichment of *Muribacter*, suggesting its association with psychological stress may be reproducible across different experimental paradigms [16]. Notably, we also detected elevated levels of *Bacteroides* in the oral cavity of CSDS mice. While *Bacteroides* are typically gut-resident commensals that contribute to health through SCFAs production, their ectopic presence in the oral niche is indicative of dysbiosis and a breakdown of normal habitat boundaries under stress [35,36]. This finding is further corroborated by studies linking the oral presence of other typically gut-associated taxa (e.g., *Prevotella*) to depression and schizophrenia, suggesting that aberrant oral-gut bacterial translocation may present a shared pathway in psychiatric disorders [37,38].

Together, CSDS induced an oral dysbiotic state characterized by the depletion of beneficial commensals and expansion of inflammation-associated or ectopic taxa. This shift likely contributes to a pro-inflammatory oral milieu, which may in turn exacerbate the systemic inflammatory state observed in depression. Although the specific taxonomic shifts vary across stress models, the overarching disruption of oral microbial ecology appears to be a consequence of stress exposure. This underscores the need for further investigation into the precise role of oral dysbiosis in the pathophysiology of depression.

Our analysis identified a distinct gut microbial signature associated with CSDS-induced depression, characterized by a shift away from healthy-associated commensals toward pro-inflammatory taxa. The gut microbiota of control mice showed significant enrichment of health-linked genera, including *Alistipes*, *Lachnospiraceae*, *Desulfovibrio*, *Erysipelatoclostridium*, *Bacteroides*, *Prevotellaceae,* and *Oscillospiraceae*. This pattern is consistent with previous reports identifying these taxa as core components of a healthy gut microbiome, with *Lachnospiraceae* in particular recognized as a dominant and beneficial bacterial group in healthy individuals [39–42].

Conversely, CSDS mice exhibited marked elevations in *Alloprevotella*, *Helicobacter*, *Muribaculum* and *Colidextribacter*. Notably, the expansion of *Alloprevotella* aligns with a previous study reporting its increase in depressive states, indicating its potential role as a microbial biomarker in stress-related disorders [43]. Similarly, the enrichment of *Helicobacter* species corresponds with their well-documented association with gastrointestinal inflammation and their emerging role in neuropsychiatric conditions through immune activation [44,45]. A particularly compelling finding was the expansion of *Colidextribacter*, a recognized pro-inflammatory pathobiont. Previous studies have correlated its abundance with both intestinal inflammation and neurochemical alterations, such as reduced GABA and homocarnosine levels in the brain, mirroring our findings and reinforcing its potential role in mediating gut-brain axis disruption in depression [46].

The CSDS-induced depression model demonstrates a clear gut microbial imbalance, marked by the depletion of beneficial bacteria and expansion of pro-inflammatory taxa. These collective changes likely contribute to gut barrier dysfunction, systemic inflammation, and neurochemical disturbances, thereby reinforcing the role of gut microbial dysbiosis in the pathophysiology of depression via gut-brain axis disruption.

The rationale for presenting oral and gut microbial data together is to identify candidate genera with potential for ectopic colonization along the oral-gut axis. For instance, *Muribacter* and *Rodentibacter* were significantly enriched in the oral cavity of CSDS mice. Interestingly, while these genera are typically considered oral-associated, their relative abundances in the gut of CSDS mice showed a similar trend (though not statistically significant). This observation raises the possibility that under chronic stress, oral pathobionts may be swallowed and survive transit through the gastrointestinal tract, potentially contributing to gut dysbiosis and subsequent brain signaling. Previous studies have demonstrated that salivary microbiota from periodontitis can induce depression-like behavior via gut microbiota modulation [47]. Our cross-habitat visualization provides a descriptive foundation for future mechanistic studies on oral-to-gut translocation in depression.

Our correlation analyses indicate that specific oral and gut microbial signatures are systematically associated with depression-like behaviors. The observed negative correlations between control-enriched taxa such as *Streptococcus-O* and *Lactobacillus-O* and behavioral deficits, coupled with the positive associations between CSDS-expanded genera (*Muribacter-O*, *Rodentibacter-O*) and such deficits, align with previous studies documenting the protective roles of commensal bacteria and the pro-inflammatory potential of pathobionts in depression [16,48–50]. These findings strengthen the link between microbial composition and behavioral alternations in stress-related disorders.

Evidence supporting the role of an oral-gut axis in depression continues to accumulate. Large-scale metagenomic analyses, such as the study by Valles-Colomer et al. have documented extensive oral-to-gut microbial transmission in humans [51]. Mechanistically, Qian et al. demonstrated that salivary microbiota from periodontitis can induce depression-like behavior through modulation of the gut microbiota [47]. In line with these findings, our study identified significant cross-habitat microbial correlations, including negative associations between health-associated and inflammation-related taxa across oral and gut ecosystems. These coordinated correlation patterns suggest potential ecological interactions between the two microbial communities and reinforce the concept of an oral-gut axis involvement in depression pathophysiology.

While the precise mechanisms underlying these interactions warrant further investigation, the identified multi-habitat microbial relationships underscore the systemic nature of dysbiosis in depression, extending beyond a single body site to encompass coordinated shifts across the oral and gut ecosystems.

## Conclusion

By integrating microbial community profiling with behavioral correlation analysis, our study delineates distinct dysbiotic patterns in both oral and gut microbiota in a model of depression and links specific microbial genera (e.g., *Muribacter*, *Rodentibacter*) to behavioral deficits via their association with pro-inflammatory taxa. These correlations establish a novel framework for understanding the systemic role of the oral-gut-brain axis in depression pathophysiology. Our findings suggest that restoring homeostasis in these interconnected microbial ecosystems, potentially by targeting key pathobionts or reinforcing beneficial commensals, may represent a promising therapeutic strategy for major depressive disorder, providing a compelling direction for future mechanistic and translational investigation.

Nevertheless, this study is subject to several limitations. First, while we observed strong correlations between microbial alterations and depression-like behaviors, these associations do not establish causality. Second, the 16S rRNA sequencing approach, though effective for taxonomic profiling, provides limited resolution for determining functional pathways and species-level differences. Third, due to difficulties in DNA extraction from mouse oral samples (low microbial biomass), our sample sizes for microbiome analysis were reduced (Oral: n = 6 per group). Fourth, our cross-sectional design provides only a snapshot of microbial alterations at a single endpoint. Longitudinal sampling during the CSDS procedure would be valuable in future studies to establish the temporal dynamics of dysbiosis and to determine whether microbial changes precede or follow the emergence of depressive-like behaviors. Despite these limitations, our findings provide compelling evidence for the involvement of both oral and gut microbiota in CSDS-induced depression, offering new insights into the microbial basis of stress-related disorders. Future studies should employ multi-omics approaches and conduct functional validation experiments to better elucidate the mechanistic links along the oral-gut-brain axis.

## Supporting information

S1 Data Raw data for Fig 1B–G and all statistical analyses presented in the manuscript. (XLSX)

S1 Fig Beta diversity of oral and gut microbiota based on Bray-Curtis distance. NMDS plots illustrating microbial community dissimilarity calculated using abundance-weighted Bray-Curtis distance. (A) Oral microbiota comparison between control (Con-O) and CSDS (CSDS-O) groups. (B) Gut microbiota comparison between control (Con-G) and CSDS (CSDS-G) groups. (C) NMDS plot showing the overall separation between oral and gut microbial communities (Con-O, CSDS-O, Con-G, CSDS-G), this panel is presented for qualitative visualization only; no direct statistical comparison was performed between oral and gut groups. O stands for oral flora, G stands for gut flora; Con stands for Healthy mice, CSDS stands for depression mice (n = 6).
(TIF)

S1 Table Summary of sequencing and microbiome data. For each group (Con-O, CSDS-O, Con-G, CSDS-G), the table presents: the range of raw and filtered reads per sample; mean ± standard deviation (SD) of ASVs per sample and genera per sample; mean Goods coverage (%); overall total ASVs and total genera across all samples in each group; and the range of Shannon diversity indices. Con, control; CSDS, chronic social defeat stress; O, oral; G, gut; ASV, amplicon sequence variant; SD, standard deviation.
(DOCX)
