# Supplementary material for: Dysbiosis of the oral-gut microbiome axis in a mouse model of depression
Source: PLoS One. 2026 Aug 11;21(8):e0355302. doi: 10.1371/journal.pone.0355302 (PMC13460603; doi:10.1371/journal.pone.0355302)
Supplement: Supplementary file 1 [file pone.0355302.s003.docx]

**Supplementary Table 1.** **Summary of sequencing and microbiome data.** For each group (Con-O, CSDS-O, Con-G, CSDS-G), the table presents: the range of raw and filtered reads per sample; mean ± standard deviation (SD) of ASVs per sample and genera per sample; mean Goods coverage (%); overall total ASVs and total genera across all samples in each group; and the range of Shannon diversity indices. Con, control; CSDS, chronic social defeat stress; O, oral; G, gut; ASV, amplicon sequence variant; SD, standard deviation.

| Group  n=6 | Raw Reads  (range) | Filtered Reads (range) | ASCs per samples (mean ± SD) | | Genera per sample (mean ± SD) | Goods Coverage  (%) |
| --- | --- | --- | --- | --- | --- | --- |
| Con-O | 65,094 – 8,0005 | 59,071 – 73,253 | | 500 **±** 40 | 216 ± 46 | 99.9 |
| CSDS-O | 49,956 – 79,984 | 46,241 – 73,738 | | 400 **±** 258 | 210 ± 124 | 99.9 |
| Con-G | 79,872 - 80041 | 73,090 – 74,944 | | 504 **±** 110 | 108 ± 16 | 99.9 |
| CSDS-G | 79,762 – 80,232 | 73,365 – 74,895 | | 503 **±** 75 | 112 ± 16 | 99.9 |

Total ASVs (all samples combined): 7,591

Total Genera (all samples combined): 927

Alpha diversity range (Shannon index): 2.11-7.12
